# Immortalising cadaveric brain dissection using application photogrammetry

**DOI:** 10.1007/s00429-026-03073-0

**Published:** 2026-02-04

**Authors:** Dearbhla P. Cullinane, Basil Lim, Denis S. Barry

**Affiliations:** 1 Discipline of Anatomy, Trinity Biomedical Sciences Institute (TBSI), Trinity College Dublin, 152–160 Pearse Street, Dublin 2, Dublin, Republic of Ireland; 2 Tisch School of the Arts, New York University, New York, USA

**Keywords:** Klingler dissection, Photogrammetry, Neuroanatomy, Medical education, Methodology

## Abstract

Cortical and deep brain topography are core components of undergraduate neuroanatomy teaching and neurosurgical training. Due to the technical challenges associated with dissecting and maintaining preserved brains, deep white matter preparations are often omitted from anatomy curricula. This reduced opportunity for tactile neuroanatomical exploration contributes to surface-level approaches to learning and is associated with poorer student outcomes including neurophobia. Photogrammetric technologies present a potential solution by offering alternate modes to learn neuroanatomy. However, these technologies often require advanced scanning and specialised resources. In this study, we present a novel approach using the Klingler dissection method to perform white matter dissections of a formalin-preserved cadaveric brain and create high-fidelity 3D models at each dissection step using a smartphone application. Lateral-to-medial and medial-to-lateral dissections were performed and high-quality 3D models were generated using Polycam. Scanning artefacts were edited in Blender, and the final models were hosted on SketchFab. A sample of models were quantitatively compared with a high-end photogrammetry pipeline and were found to maintain exceptional visual and structural fidelity. The preservation of neuroanatomical dissections in this manner has the potential to improve resource accessibility, enhance spatial understanding, and guide the development of low-cost transferrable teaching resources.

**Supplementary Information:**

The online version contains supplementary material available at 10.1007/s00429-026-03073-0.

## Introduction

A decrease in the use of cadavers for anatomical education and institutional regulations have led to a reduction in structural white matter dissections of cadaveric brains (Newman et al. 2021; Sotgiu et al., 2020). However, an in-depth knowledge of neuroanatomy is becoming more relevant as all physicians begin to treat ageing and more varied populations (Henderson et al. 2023; Tan et al. 2021). For neurosurgical trainees, developing a thorough mental representation of subcortical anatomy is essential for minimising the risk of postoperative deficits and should not be undervalued (Bozkurt et al. 2016; De Benedictis et al. 2018; Maldonado 2024). Moreover, enhanced understanding of white matter tracts can provide insights into neuronal communication, general brain function, and the underlying neural mechanisms thought to be implicated in various neurological and neuropsychiatric disorders including dementia, schizophrenia, Parkinson’s disease, and functional neurological disorder (Baykara et al. 2022; Butt et al. 2021; Chen et al. 2023; Lee et al. 2015; Medina et al. 2021; Tan et al. 2020).

Despite the advent of multimedia tools, students are often unable to achieve a clear understanding of the spatial relationships that exists within the internal topography of the brain (Silva and Andrade 2016). This has led to the widespread perception that neuroanatomy and the neurosciences are inherently difficult to teach and learn (Javaid et al. 2018; Jozefowicz 1994). One approach to addressing this challenge is the use of the Klingler technique (Dziedzic et al. 2021; Silva and Andrade 2016); a modified method of brain fixation and dissection based on freezing and thawing of brain tissue, subsequent peeling away of grey matter, and the gradual exposure of white mater tracts (Wysiadecki et al. 2019). The stratigraphic manner in which the technique is applied is potentially fundamental in understanding gross subcortical architecture; however, the process is complex and technically challenging. As such, in conjunction with low specimen availability, increasing student numbers, and decreased curricular contact time, virtual representations of neuroanatomical material has been presented as a scalable solution (de Oliveira et al. 2023).

Photogrammetry refers to the process of taking several two-dimensional (2D) photographs and stitching them together to form a 3D reconstruction (Krause et al. 2023). This is achieved by calculating the relative position of common points in subsequent images of a subject (Erolin 2019), with further accuracy attainable through the integration of point cloud data derived from LIDAR scanning (Habib et al. 2004). A recent review of its applications in anatomical education suggests that photogrammetry holds significant promise as a teaching tool (Chytas et al. 2024). Until recently, however, it has been understood that state-of-the-art digital equipment was essential for obtaining real-world fidelity and anatomical accuracy (De Benedictis et al. 2018; de Oliveira et al. 2023; Hanalioglu et al. 2022; Krause et al. 2023; Nocerino et al. 2017). However, emerging evidence indicates that open source photogrammetry software (Van Vlasselaer et al. 2024) and smartphone devices may be able to achieve equivalent quality and precision (Krogager et al. 2023; Morichon et al. 2024), particularly if equipped with LIDAR capability. This shift raises an important pedagogical question: can low-cost technologies replicate the realism and anatomical diversity typically encountered in dissection theatres, and are they equivalent to more technologically advanced systems?

With recent progresses and growing adoption for photogrammetry across the anatomical sciences, this technology appears particularly well suited to addressing the challenges of teaching and learning neuroanatomy (Chytas et al. 2024). This study presents a novel integration of two established approaches, white matter dissection and smartphone photogrammetry, and offers both a qualitative and quantitative assessment of geometric fidelity for this photogrammetry pipeline. It demonstrates the transferability of an adapted Klingler technique alongside low-cost, scalable, and user-friendly photogrammetric scanning and post-processing methods. While each technique has proven valuable independently, their combined application in this context and comparison with a high-end pipeline represents a new and practical strategy for enhancing neuroanatomical education.

## Materials and methods

### Sample Preparation

The brain and dura mater were extracted from the donor’s cranium before immersion in 10% formalin solution for six months. The brain was washed under fresh cold water and transferred to a cold-water container for 24 h. The dura mater was then removed and the caudal brainstem and cerebellum were detached from the diencephalon. Subsequently, the arachnoid membrane and meningeal vessels were removed from the cerebrum using a non-toothed forceps and microsurgical scissors. After the external topography of the cerebrum had been inspected for macroscopic pathology, the brain was sectioned through the sagittal sulcus using a brain knife and brain-sectioning apparatus. All remaining arachnoid mater was removed from the medial surface of each hemisphere. Both hemispheres were placed into individual plastic freezer bags, labelled, and stored in a chest freezer at -17 °C for two weeks.

While enclosed within their respective plastic freezer bags, each hemisphere was removed from the chest freezer and thawed under cold running water for 4 h. The cerebral hemispheres were stored in a 2.5% formalin solution between dissection sessions. To facilitate dissection, hemispheres were re-frozen and thawed as required.

### Dissection procedure

A dissection protocol by Koutsarnakis et al. (2015) was adapted for this study. Two dissection approaches were performed: lateral-medial dissection and medial-lateral dissection. High quality photographs using a Canon EOS RP camera with a 24–105 mm f/4-7.1 IS STM lens and 3D photogrammetry scans using an Apple iPhone 12 (with a standard 12 MP rear camera) were taken after each step of dissection. The Canon camera was set to automatic capture mode to imitate the automatic capture setting used by the photogrammetric mobile phone application.

#### Lateral-Medial dissection (Left Hemisphere)

**Fig. 1:**
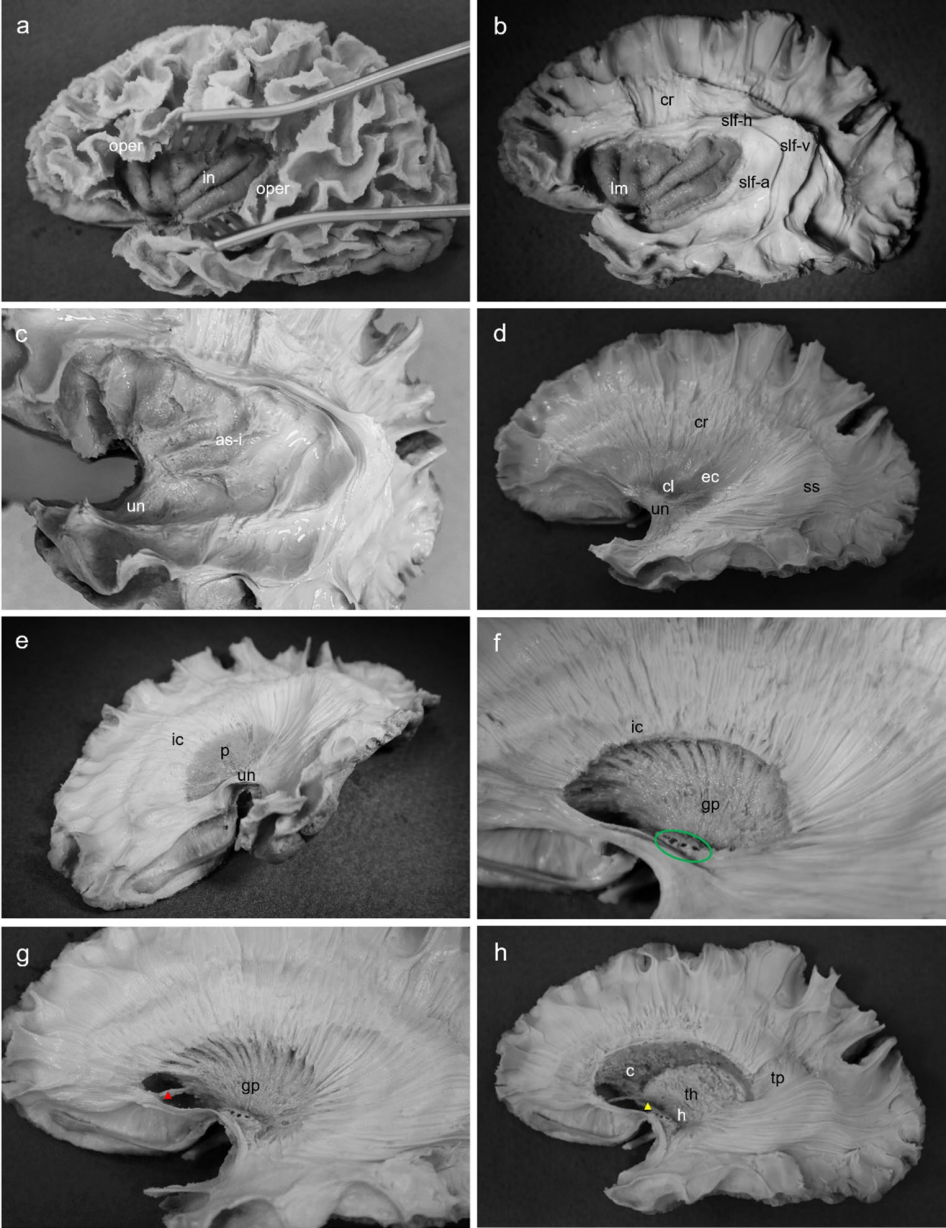
Lateral-to-medial stepwise dissection of left cerebral hemisphere. Procedure adapted from Koutsarnakis et al. (2015). **a**, Lateral cerebral cortex removed, insular cortex visible by retracting the operculum; **b**, operculum removed and superior longitudinal fasciculus exposed; **c**, magnified view of insular association fibres; **d**, superior longitudinal fasciculus removed, claustrum and uncinate fasciculus visualised; **e**, rostro-ventral view of lateral cortex, claustrum and external capsule removed to expose the putamen; **f**, magnified lateral view of diencephalon, putamen removed to expose globus pallidus; **g**, substantia innominata removed to expose anterior fascicle of anterior commissure; **h**, globus pallidus removed to expose thalamus and posterior of anterior commissure. as-i, insular association fibres; c, caudate nucleus; cl, claustrum; cr, corona radiata; ec, external capsule; gp, globus pallidus; h, hypothalamus; ic, internal capsule; in, insula; lm, limen insula; oper, operculum; p, putamen; slf-a, superior longitudinal fasciculus arcuate segment; slf-h, superior longitudinal fasciculus horizontal segment; slf-v, superior longitudinal fasciculus vertical segment; ss, sagittal stratum; th, thalamus; tp, tapetum; un, uncinate fasciculus. The green circle indicates the lenticulostriate arteries. The red triangle indicates the anterior fascicle of the anterior commissure. The yellow triangle indicates the posterior fascicle of the anterior commissure

1. Beginning at the depth of the superior temporal sulcus and extending along the entire lateral surface of the cerebrum, the cerebral cortex was removed using a blunt bent-end surgical probe and non-toothed forceps. The bent-end probe was placed into the depth of the superior temporal sulcus. Downward pressure was applied to allow the probe to pass through the substance of the cerebral cortex. On meeting resistance, the white matter was reached, and using a lifting effect, the probe was titled laterally to detach cerebral cortex from underlying white mater. Association fibres were visualised, and the operculum was left in-tact to demonstrate the relationship between the insula and its lateral coverings (Fig. 1a). The hemisphere was re-frozen for 15 h to generate additional inter-axonal lacunas and underwent defrosting for 4 h.
2. Dissection continued by removing the association fibres to expose the superior longitudinal fasciculus (SLF) which extended from the frontal to temporal lobe. A small vertical window was made in the rostral section of the SLF to expose projections of the corona radiata and to showcase the width of the SLF. The insular cortex was exposed by removing the operculum using a micro-surgical scissors (Fig. 1b).
3. The insular cortex was then removed to reveal the insular association fibres. The rostral insular- temporal connection was identified as the uncinate fasciculus (Fig. 1c).
4. Starting at the depth of the corona radiata window, the remaining SLF was removed. Removal of the SLF in the temporo-occipital region facilitated visualisation of the sagittal stratum. At the insula, association fibres were dissected away to expose the external capsule and claustrum. The claustrum appeared as a thin layer of grey matter closely adherent to the external capsule (Fig. 1d).
5. Careful removal of the external capsule using a non-toothed forceps exposed the putamen. The integrity of the uncinate fasciculus rostrally was maintained (Fig. 1e).
6. The putamen, which was spongy in consistency, was dissected away to reveal the globus pallidus deep to it. The globus pallidus was firm in consistency and distinguishable from the putamen. At the dorsal end of the globus pallidus the root of the lenticulostriate arteries was also exposed (Fig. 1f).
7. Delicate dissection rostral to the globus pallidus removed the substantia innominata of the basal forebrain leaving behind the anterior commissure (anterior fascicle). At the ventral aspect of the globus pallidus, white mater was removed to reveal the gradual merging of grey mater to white mater at the internal capsule (Fig. 1g).
8. The remainder of the internal capsule and globus pallidus were removed exposing the thalamus, hypothalamus, and head, body, and ventral tail of the caudate nucleus. The consistency of the caudate was similar to the putamen and was easily identifiable. The thalamus and hypothalamus had a firm consistency similar to that of the globus pallidus. Rostral to the hypothalamus, the anterior commissure (posterior fascicle) gradually became more visible. Lastly, a small oblique window was dissected superior to the sagittal stratum to reveal perpendicular fibres of the tapetum (Fig. 1h).

#### Medial-lateral dissection (right hemisphere)

**Fig. 2:**
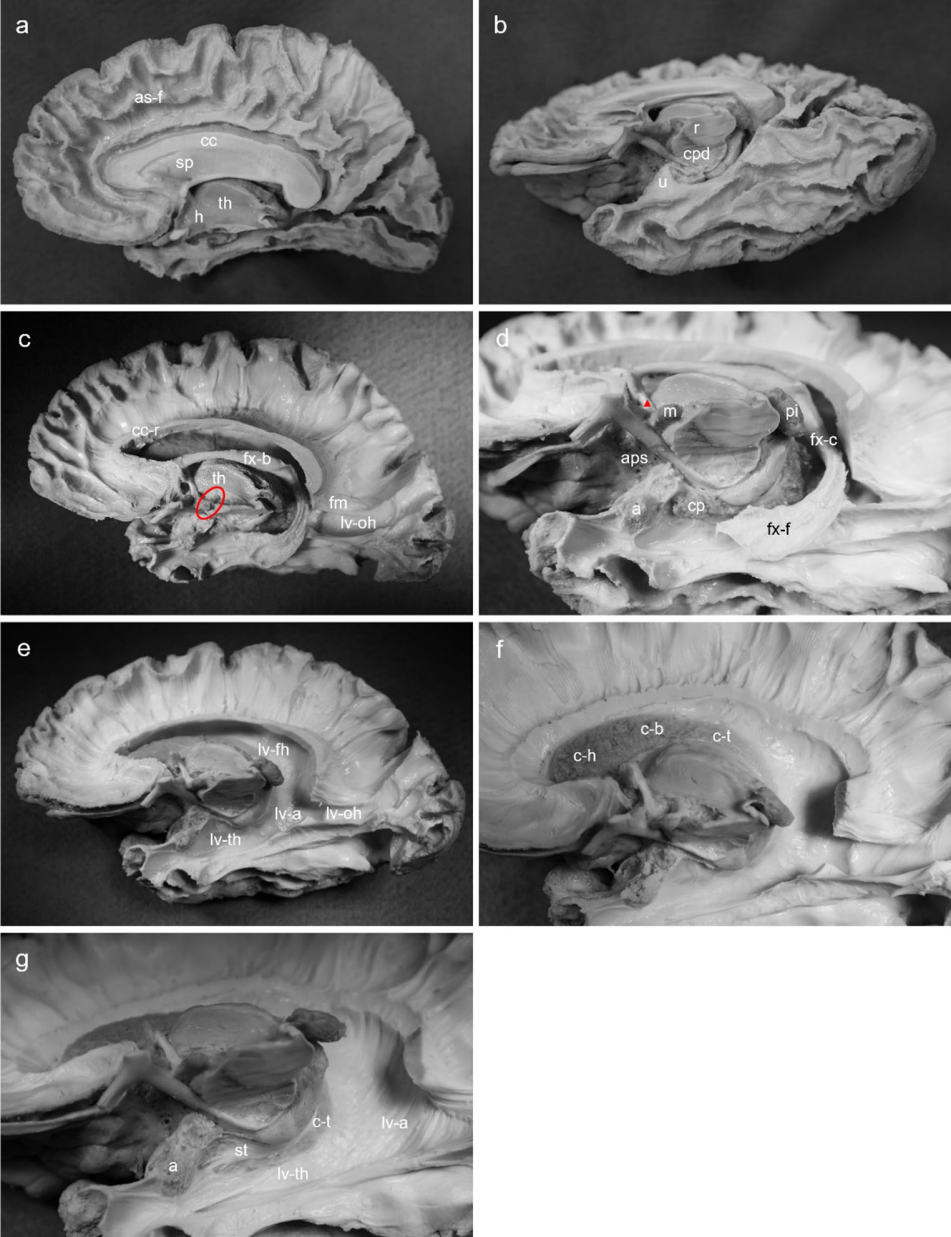
Medial-to-lateral stepwise dissection of right cerebral hemisphere. Procedure adapted from Koutsarnakis et al. (2015). **a**, mid-sagittal view of right hemisphere with cerebral cortex removed and association fibres of frontal, parietal, and occipital lobes visible; **b**, inferior view of right hemisphere with cerebral cortex removed and association fibres of temporal lobe visible; **c**, mid-sagittal view of right hemisphere with association fibres removed, corpus callosum resected, and hippocampal formation and mammillothalamic tract exposed; **d**, magnified inferior view of right hemisphere showcasing the extent of the hippocampal fimbria, choroid plexus, and amygdala; **e**, mid-sagittal view of right hemisphere with hippocampal formation and choroid plexus removed; **f**, magnified mid-sagittal view of right hemisphere with corpus callosum fully resected and ependymal covering on head and body of caudate nucleus removed; **g**, magnified anterior-inferior view of right hemisphere with stria terminalis fibres visible immediately rostral to the tail of the caudate nucleus. **a**, amygdala; aps, anterior perforating substance; as-f, frontal association fibres; as-t, temporal association fibres; c-b, body of caudate nucleus; c-h, head of caudate nucleus; c-t, tail of caudate nucleus, cc, corpus callosum; cc-r, resected corpus callosum; cpd, cerebral peduncle; fm, forceps major; fx-b, body of fornix; fx-c, crus of fornix; fx-f, fimbria of fornix; h, hypothalamus; lv-a, atrium of lateral ventricle; lv-fh, frontal horn of lateral ventricle; lv-oh, occipital horn of lateral ventricle; lv-th, temporal horn of lateral ventricle; m, mammillary body; pi, pineal gland; r, red nucleus; sp, septum pellucidum; st, stria terminalis; th, thalamus; u, uncus. The red circle indicates the mammillothalamic tract. The red triangle indicates the anterior column of the fornix

1. Using a no.15 scalpel blade, the midbrain was removed from the diencephalon by making a cross-sectional incision starting at the depth of the inter-peduncular fossa rostrally and continuing to the superior colliculus caudally. The cerebral cortex was then removed using a blunt bent-end surgical probe and non-toothed forceps, starting at the depth of the anterior cingulate gyrus, and continuing posteriorly along the entire medial surface to the precuneus (Fig. 2a). Caution was taken to reach the depth of the parietooccipital sulcus before continuing into the occipital and temporal lobes (Fig. 2b). All association fibres from the medial aspect of the cerebrum were then exposed.
2. The corpus callosum and septum pellucidum were resected using a no.15 scalpel blade leaving a thin strip of colossal fibres attached to the cingulum dorsally and the body of the fornix ventrally. The ventricular ependyma, choroid plexus and caudate nucleus were visualised in the superior horn of the lateral ventricle. Beginning at the parietal portion of the corpus callosum, the thin strip of colossal fibres were followed dorsally to remove the association fibres from the medial surface of the cerebrum. Caudally, caution was taken to preserve the forceps major. Ventral to the forceps major, the occipital horn of the lateral ventricle was entered before continuing rostrally to reveal the temporal horn (Fig. 2c). The hypothalamus was then inspected immediately ventral to the mammillary body. Careful teasing of grey mater towards the hypothalamic sulcus exposed the mammillothalamic tract running ventral-caudal from the mammillary body towards the thalamus. Likewise, the anterior column of the fornix was identified and then followed ventrally to the mammillary body. The crus of the fornix was followed ventral-caudally into the hippocampal formation of the temporal lobe. Using a blunt probe the fimbria of the fornix was made visible by teasing away the substance of the hippocampus. Rostrally, the uncus was dissected using a non-toothed forceps until the spongy appearance of the amygdala was reached (Fig. 2d).
3. Using a no.15 scalpel blade, an incision was made through the fornix at the junction of the body and anterior column. The choroid plexus of the lateral ventricle was teased away using a non-toothed forceps. By removing the fornix and choroid plexus, the full extent of the lateral ventricle was visualised (Fig. 2e).
4. The remaining strip of corpus callosum was dissected to demonstrate the extent and direction of colossal radiations yet leaving the forceps major in place. Ventricular ependyma was then gently peeled from the head and body of the caudate nucleus. The ependyma of the tail was kept in place to distinguish the change in appearance of the caudate nucleus (sponge-like) and its ependymal covering (glossy) (Fig. 2f).
5. Finally, the remainder of the caudate nucleus was exposed by removing the ependymal layer from the atrium of the lateral ventricle and continuing into the occipital and temporal horns. Medial and rostral to the tail of the caudate nucleus, the stria terminalis and its projections to the amygdala were revealed (Fig. 2g).

### Generating 3D models

#### 3D scanning protocol

**Fig. 3:**
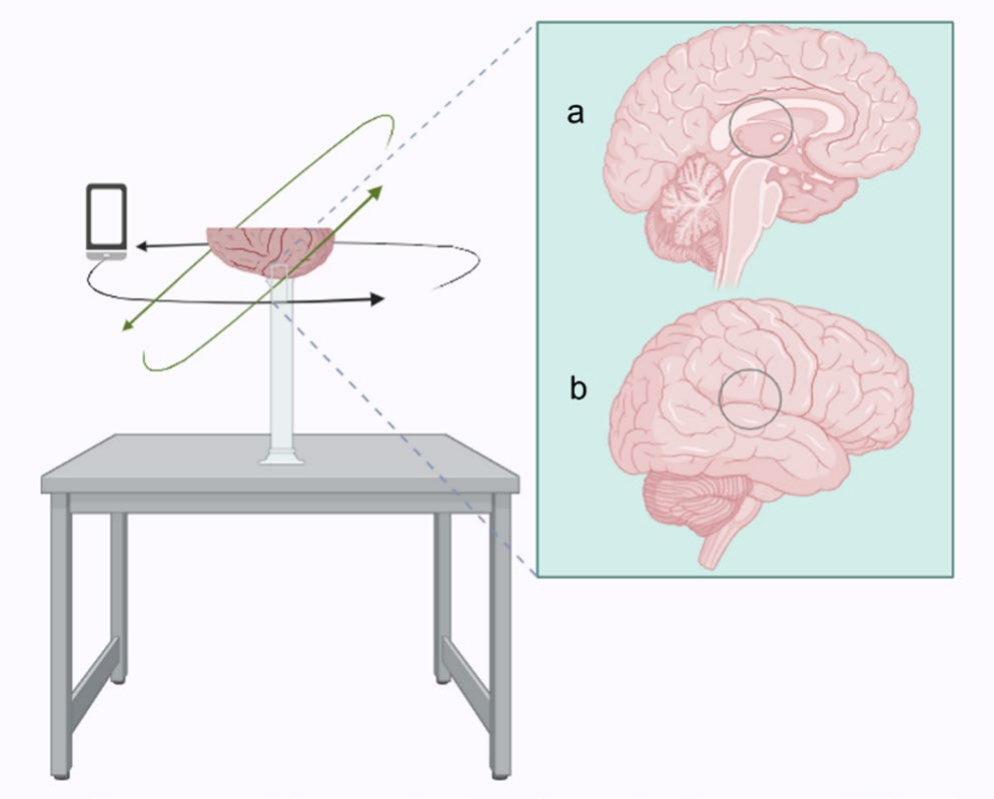
Schematic showing scanning apparatus. The setup includes a workbench, small, graduated cylinder, and an iPhone 12 with Polycam mobile application. The scanning object remains stationary while the operator circumnavigates the scanner (iPhone) around the specimen. A continuous series of photographs are obtained. **a**, circle indicates approximate gravitational centre for left hemisphere; **b**, circle indicates approximate gravitational centre for right hemisphere. Created in https://BioRender.com

At the end of each dissection step, the specimen was dried using paper towels and placed on a small, graduated cylinder to elevate the specimen from the workbench and facilitate 360° scanning (Fig. 3). The lateral-to-medial dissected hemisphere was placed with the mid-sagittal surface facing inferiorly towards the workbench. The gravitational centre comprised the thalamus and the caudal body of the corpus callosum (Fig. 3a). The medial-to-lateral dissected hemisphere was placed with the lateral surface facing inferiorly. The gravitational centre of the lateral surface was approximately 5 cm rostral to the angular gyrus at the level of the lateral sulcus (Fig. 3b).

**Fig. 4:**
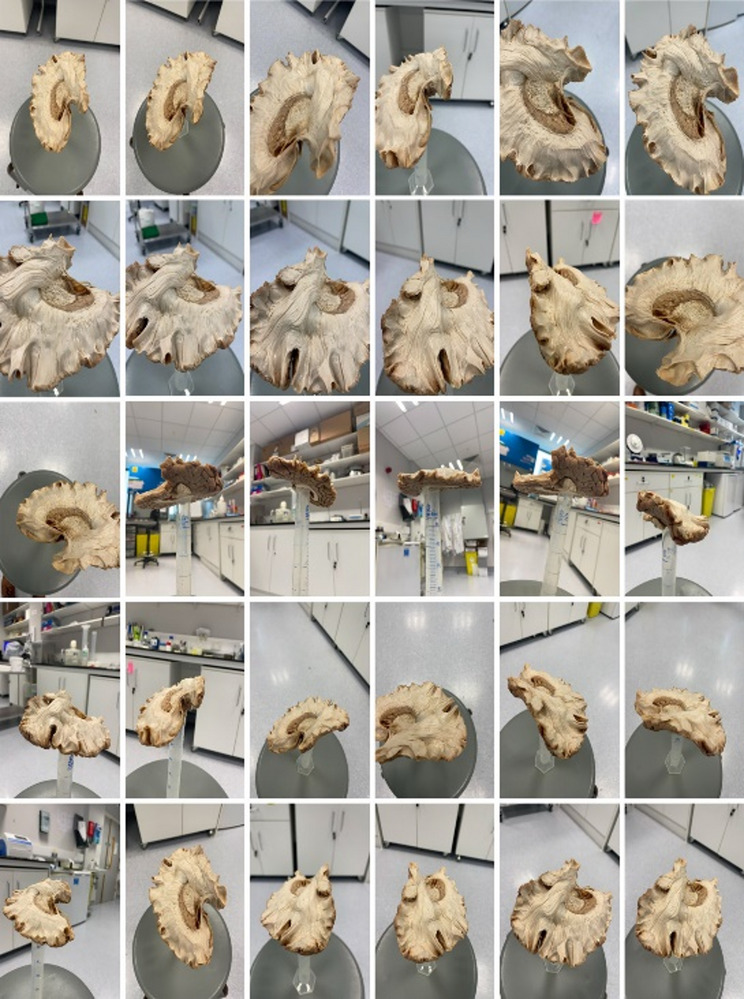
Sample of 30 iPhone images acquired during video mode capture in the Polycam application. A variety of perspectives, focal depths, and scales are obtained while circumnavigating the specimen. Images were part of the reconstruction input for lateral-medial dissection step 8. Total number of images used to generate the final model was *N* = 173. A link to the final 3D model can be seen in Table 1

Circulating the brain, a continuous and automated series of photographs were taken under object video mode using the 3D scanning application Polycam (Polycam Inc. 2023). Photographs were taken from several perspectives, focal depths, and scales to ensure comprehensive coverage of the specimen. A sample of 30 images from one of the thirteen scanning procedures can been seen in Fig. 4. Video mode automatically captures one to two photographs per second with a 30% to 50% overlap between frames. Each scan consisted of approximately 170 to 200 images. The lighting source was provided by fixed overhead laboratory lighting which permitted as consistent and even an illumination of the specimen as possible. A dedicated light-controlled enclosure such as an illumination box was not available.

#### Post-scanning processing and editing

After scanning, images were visually inspected on the Polycam application. Images with significant blurring or artefacts were removed from the draft file. The detail setting was set as ‘raw’ so as to preserve anatomical clarity and retain access to original .*heif* files. Processing occurred via Polycam’s cloud server to allow for rapid modelling. The 3D models generated were automatically uploaded to a private Polycam library which could then be accessed from a laptop or PC. Basic cropping of visual artefacts, including superior aspects of the graduated cylinder were cropped from the original mesh models using the online Polycam editor. Mesh models were then exported as *FBX* files.

FBX files were imported to Blender, an open-source 3D modelling program (Blender 2024). Polycam 3D mesh files were interpreted by Blender in smaller units, requiring manual scaling adjustments via the transform pop-out window, thus models were uniformly scaled by a factor of 100, normalising object scale values from 0.01 to 1.0. Once scaling was completed, the 3D model was edited by switching from object to edit mode. Blender’s advanced cropping tools allowed for precise removal of artefacts from several angles and shapes, a capability not available through the Polycam editing tool. Origin points were selected whilst in edit mode and moved to central portions of the model. Models were then re-exported as FBX and subsequently imported to SketchFab.

### Analytical approach

A qualitative visual assessment was conducted on 2D images and photogrammetric reconstructions to identify artefacts such as reflections, shadowing, and overexposure. These assessments were complemented by quantitative analyses of the 3D reconstructions, including mesh density and cloud-to-cloud comparisons, to evaluate reconstruction quality and geometric consistency. The computerised procedures for quantitative assessment are provided in Supplementary File 1.

## Results

### Visual comparison of camera and phone images with Polycam reconstructions

**Fig. 5:**
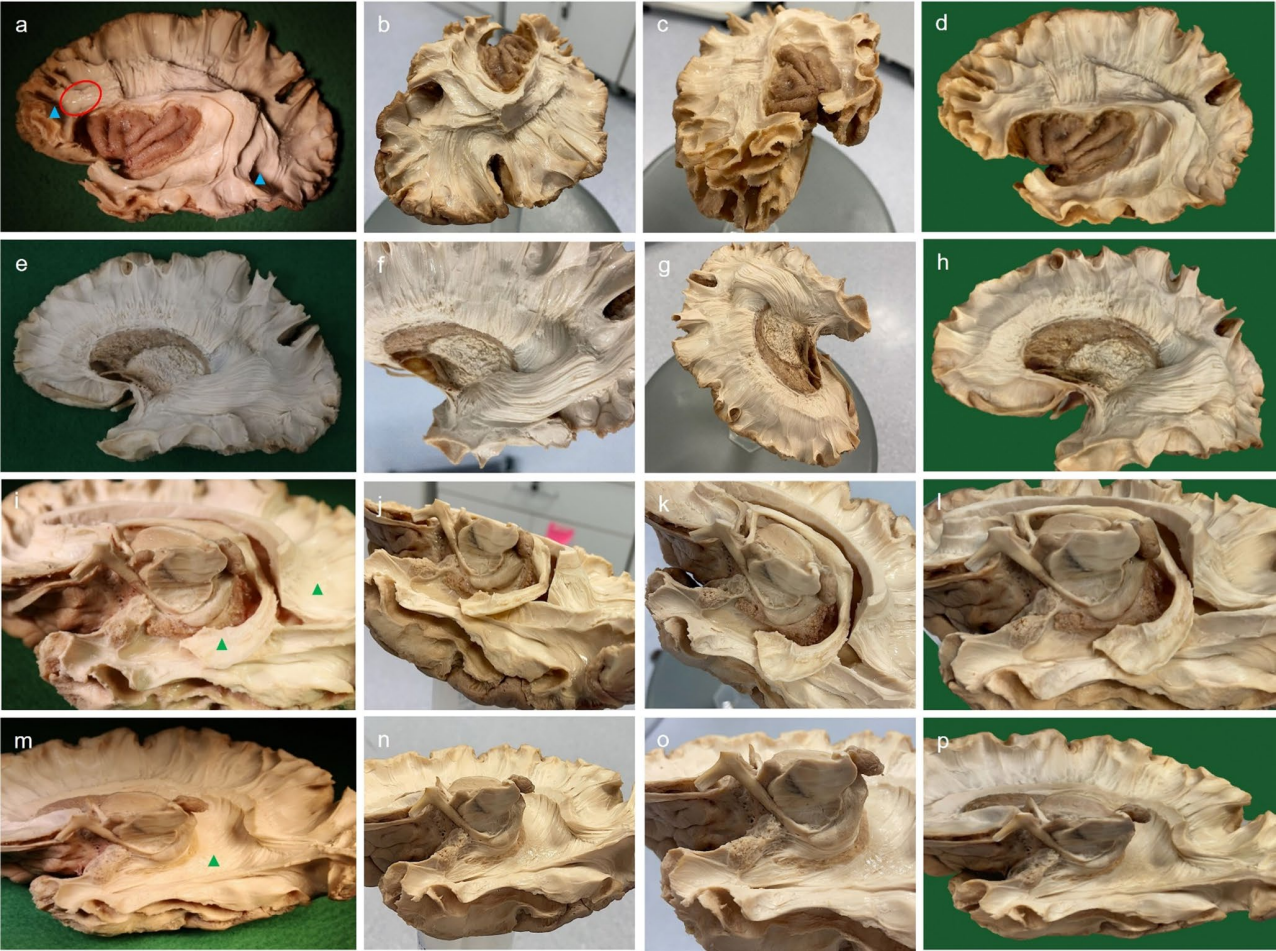
Side-by-side comparisons of camera images, phone images (x2), and screenshots of photogrammetric reconstructions. **a**, camera image of specimen as per lateral-medial dissection step 2; **b** and **c**, phone images lateral-medial dissection step 2; **d**, screenshot of photogrammetric reconstruction lateral-medial dissection step 2; **e**, camera image of specimen as per lateral-medial dissection step 8; **f** and **g**, phone images lateral-medial dissection step 8; **h**, screenshot of photogrammetric reconstruction lateral-medial dissection step 8; **i**, camera image of specimen as per medial-lateral dissection step 2; **j** and **k**, phone images medial-lateral dissection step 2; **l**, screenshots of photogrammetric reconstruction medial-lateral dissection step 2; **m**, camera image of specimen as per medial-lateral dissection step 5; **n** and **o**, phone images medial-lateral dissection step 5; **p**, screenshot of photogrammetric reconstruction medial-lateral dissection step 5. Red circle, light is reflected due to the combined use of over-head surgical lighting and insufficiently dried specimen; blue triangles, peripheral shadowing; green triangles, overexposure causing excessive brightness

2D images captured using the Canon camera exhibited several visual artefacts (Fig. 5). These included light reflections from specimen that were insufficiently dried (Fig. 5a), peripheral shadowing (Fig. 5m), and overexposure resulting in excessive brightness and a loss of finer details (Fig. 5i). These issues persisted despite efforts to simulate a studio-like photography environment. An alternative setup utilised the fixed overhead laboratory lighting in lieu of surgical lighting. While this approach eliminated peripheral shadowing, minimised overexposure, and evenly illuminated the whole specimen, it resulted in reduced image quality (see Fig. 5e). When capturing images with the Canon camera, the shooting mode was set to automatic to replicate the automatic capture mode facilitated by the Polycam application.

The original texture and colour of the dissected surfaces were well-preserved in the Polycam models. Reflections and overexposure were automatically processed out, and no distortions were observed along the peripheral edges of the models (Fig. 5d h, l, p).

### Processing artefacts

**Fig. 6:**
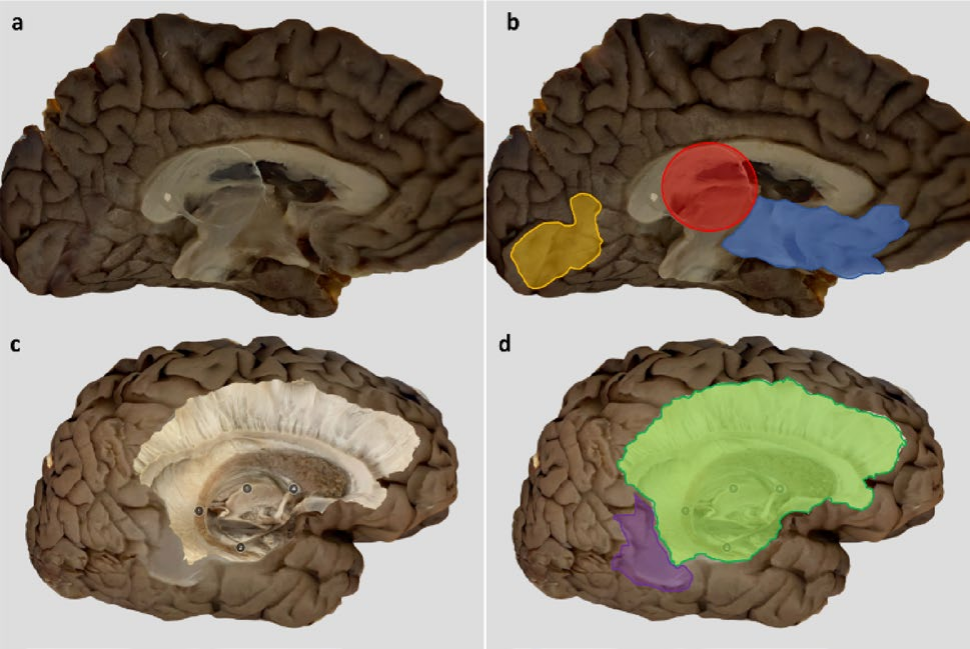
Scanning artefacts: **a**, non-dissected medial side of left hemisphere as showcased in Sketchfab viewing platform; **b**, non-dissected medial side of left hemisphere with artefacts indicated; **c**, non-dissected lateral side of right hemisphere as showcased in Sktechfab viewing platform.; **d**, non-dissected lateral side of right hemisphere with artefacts indicated. Red circle, signifies site of gravitational centre for placement of graduated cylinder; blue shape, indicates blurred artefact at diencephalon and basal forebrain; yellow shape, indicates blurred artefact at occipital-temporal junction; green shape, indicates portion of the lateral surface where cropping was required to remove all blurred artefacts; purple shape, indicates remaining blurred artefact at middle temporal gyrus

The non-dissected surface, which faced inferiorly towards the workbench, displayed several blurred artefacts that could not be corrected (see Fig. 6). For example, on the medial surface of the left hemisphere (lateral-to-medial dissections), three consistent artefacts were observed. The first was a circular remnant at the gravitational centre caused by the graduated cylinder used to prop-up the specimen during scanning. Additionally, the position of the specimen resulted in two artefacts likely due to limited access for close-range scanning of the underside. This resulted in a blurring of the rostral diencephalon and basal forebrain, as well as a defect in visual clarity at the medial occipitotemporal junction (see Fig. 6b). On the lateral surface of the right hemisphere (medial-to-lateral dissections), a larger artefact was identified, most of which was cropped out during post-scanning editing (see Fig. 6d).

### Accessing and navigating the 3D models

The final 3D scans were labelled and housed on Sketchfab, a free online platform for viewing and interacting with 3D models. Sketchfab URLs are paired with corresponding 2D dissection images for reference (Table 1). Accompanying descriptions are navigational and designed to guide viewers through key features and landmarks, facilitating interactive and self-directed exploration of the 3D models. 

**Table 1:** List of 3D models and their associated Sketchfab urls

Model name, dissection step	Accompanying anatomical description	Sketchfab URL	Corresponding figure(s)
Lateral View			
Lateral I, Step 1	The first step is to remove the cerebral cortex from the lateral surface of the cerebrum starting at the depth of the superior temporal sulcus. The superior temporal sulcus is label number 1 on this scan and is the superior-most groove on the lateral temporal lobe. Labels 2, 3, and 4 are the frontal, parietal and temporal segments of the operculum respectively. They protrude into and surround the lateral sulcus, forming a lid that covers the insula. Labels 6, 7, 8, and 9 are the association fibres of the frontal, parietal, temporal, and occipital lobes. They are exposed after the grey matter of the gyri and sulci are removed.	https://sketchfab.com/3d-models/lateral-i-607904afbca44d3c8bdfbbc3c0960f9e	Fig. 1a
Lateral II, Step 2	The second step is to remove the operculum to reveal the insular cortex. The ‘U’ fibres are also removed to expose the superior longitudinal fasciculus, which is label 1 on this scan. The superior longitudinal fasciculus is a large fibre bundle that interconnects the frontal lobe with the parietal and temporal lobes. Its vertical segment, the segment between the parietal and temporal lobes, is labelled as label 2. Label 3 represents the arcuate portion of the superior longitudinal fasciculus an connects Broca’s and Wernicke’s areas in the dominant hemisphere. Label 4 shows the limen insula, the most anteroinferior aspect of the insula, and label 5 shows the central insular sulcus, the groove at the centre of the insula that separates the short gyri from the long gyri. Label 6 is the corona radiata, a white matter sheet containing ascending and descending axonal bundles which continues inferiorly as the internal capsule and superiorly as the centrum semiovale.	https://sketchfab.com/3d-models/lateral-ii-e99c4f66fec94076a058419cfc4f3a81	Fig. 1b
Lateral III, Step 3	The third step is to peel away the insular cortex to expose the insular association fibres. At this level of dissection, the association fibres and the uncinate fasciculus constitute part of the extreme capsule. On this scan, the association fibres of the superior long gyrus is labelled as label 1. Label 2 is the ventral aspect of the uncinate fasciculus, a white matter tract that connects the orbitofrontal cortex to the anterior temporal lobe. The exact function of the uncinate fasciculus is still debated but is thought to be involved in language, trial and error learning, and pairing names with faces.	https://sketchfab.com/3d-models/lateral-iii-c2f1919f65174f038177522a5cc3cf3e	Fig. 1c
Lateral IV, Step 4	The fourth step is to remove the fibres of the extreme capsule to reveal the uncinate fasciculus more clearly and to expose the external capsule and the claustrum. On this scan, label 1 is the claustrum. The claustrum is a thin sheet of grey matter between the striatum and the insular cortex. Label 2 is the external capsule, which consists of white matter tracts lateral to the putamen and deep to the claustrum. Label 3 is the corona radiata and label 4 is the sagittal stratum, otherwise known as the optic radiation. The sagittal stratum connects the lateral geniculate nucleus of the thalamus to the primary visual cortex. Label 5 is the uncinate fasciculus, a white matter tract that connects the orbitofrontal cortex to the anterior temporal lobe.	https://sketchfab.com/3d-models/lateral-iv-bfe8aac2a37c47cba68f461fdd30018a	Fig. 1d
Lateral V, Step 5	The fifth step is to remove the remaining parts of the superior longitudinal fasciculus and the fibres of the external capsule to reveal the putamen and the internal capsule. Label 1 shows the lateral putamen. The putamen is a round structure of the basal ganglia and is lateral to the globus pallidus. It is part of the striatum along with the caudate nucleus and the globus pallidus. Label 2 is the internal capsule, a white matter sheet containing ascending and descending axonal bundles. Here, it is blending with the corona radiata dorsally. Label 3 is the uncinate fasciculus.	https://sketchfab.com/3d-models/lateral-v-15fe17a65a41497c82dc31a8de732c8d	Fig. 1e
Lateral VI, Step 6	The sixth step is to dissect the putamen to reveal the globus pallidus, which is label 1 on this scan. The globus pallidus is a component of the lentiform nucleus that is medial to the putamen. Label 2 shows four lenticulostriate arteries, which arise from the M1 segment of the middle cerebral artery and supply the deep brain nuclei. Label 3 indicates the substantia innominata. The substantia innominata has many other names including the basal forebrain and the anterior perforating substance. It is an area of mixed grey and white matter that has a high concentration of cholinergic neurons.	https://sketchfab.com/3d-models/lateral-vi-ff4dac3c2c1146fc9fb7d52cf341d295	Fig. 1f
Lateral VII, Step 7	The seventh step is to remove the substantia innominata to expose the anterior commissure. Label 1 shows the anterior fascicle of the anterior commissure. This commissure is transversely oriented and connects the orbitofrontal cortices of the two cerebral hemispheres. Label 2 is the globus pallidus, a structure of the basal ganglia medial to the putamen. Label 3 shows the globus pallidus blending into the internal capsule.	https://sketchfab.com/3d-models/lateral-vii-9d76ed6145bb4e92a9d90edc05ada67c	Fig. 1g
Lateral VIII, Step 8	The last step is to gradually remove the globus pallidus and the remainder of the internal capsule to expose the caudate nucleus and the thalamus. Labels 1, 2, and 3 annotate the head, body, and tail of the caudate nucleus respectively. The caudate nuclei are bi-hemispheric C-shaped structures of the basal ganglia located lateral to the lateral ventricles. Label 4 is the thalamus, and immediately ventral to the thalamus is label 5, the hypothalamus. Gradual removal of the globus pallidus brings the posterior fascicle of the anterior commissure into view, labelled number 6 on this scan. The posterior fascicle of the anterior commissure passes caudally before dividing into temporal and parieto-occipital divisions. The temporal division terminates in the amygdala. The dorsal portion of the optic radiation was carefully removed to reveal the tapetum deep to it. The tapetum, label 7 on this scan, is fibre bundle within the corpus callosum that extends along the lateral surface of the occipital and temporal horns of the lateral ventricle. Label 8 is the amygdala. Labels 9 and 10 are the olfactory bulb and the olfactory tract respectively. Label 11 is the anterior perforating substance, an area of mixed grey and white matter that has a high concentration of cholinergic neurons and is perforated by lenticulostriate arteries. Labels 12, 13, and 14 are the optic nerves, optic chiasm, and the optic tracts respectively.	https://sketchfab.com/3d-models/lateral-viii-9933a67d27334be28fc47a0a299b84c5	Fig. 1h
Medial View			
Medial I, Step 1	The first step is to remove the cerebral cortex from the medial surface of the cerebrum. In doing so, the association fibres become visible. Labels 1–4 show the frontal, parietal, temporal, and occipital association fibres respectively. Label 5 indicates the corpus callosum which is the largest white matter tract connecting the right and left cerebral hemispheres. Immediately ventral to the corpus callosum is a thin, translucent mixed white and grey matter sheet called the septum pellucidum. The septum pellucidum separates the frontal horns of the lateral ventricles and is attached to the fornix ventrally. Label 7 indicates the body of the fornix on this scan. Below the fornix is the thalamus, a large mass of grey matter, and rostro-ventral to the thalamus is the hypothalamus at label 9. Label 10 shows the optic chiasm from the mid-sagittal view. Moving caudally, the full depth of the parieto-occipital sulcus and calcarine sulcus can be appreciated at labels 11 and 12 respectively.	https://sketchfab.com/3d-models/medial-i-e8f8ee1d7c7b40c693e4b3b8782d933e	Fig. 2a and b
Medial II, Step 2	The second step is to resect the dorsal aspect of the corpus callosum, remove the septum pellucidum, and expose the fornix. Label 1 shows the centrum semiovale. The centrum semiovale is the superior continuation of the internal capsule containing ascending and descending axonal bundles. Label 2 indicates the remaining inferior boarder of the corpus callosum. The dorsal portion has been resected. The anterior column of the fornix is indicated by label 3. This is made visible by removing some of the ependymal layer from the third ventricle. Moving caudally, label 4 indicates the crus of the fornix, and following the tract ventrally, the crus fans out to become the fimbria at label 5. Rostral to the anterior column of the fornix is the anterior commissure at label 6. Immediately ventral to the thalamus, the substance of the hypothalamus has been removed to expose the mammillothalamic tract at label 7. The mammillothalamic tract carries signals from the mammillary bodies to the anterior thalamus and functions to support spatial memory. The mammillary body is labelled number 8 on this scan. Moving caudally, the pineal gland is label 9, and the choroid plexus is label 10. The amygdala is indicated at label 11.	https://sketchfab.com/3d-models/medial-ii-e3090803e41a4c4ab9a896ee1dd01f62	Fig. 2c and d
Medial III, Step 3	The third step is to remove the fornix, enabling us to see the full extent of the lateral ventricle. Labels 1–3 indicate the frontal, temporal, and occipital horns respectively. The point at which all three horns converge is called the atrium of the lateral ventricle and is indicated at label 4. Contributing to the roof of the occipital horn is the forceps major at label 5. The forceps major is a u-shaped white matter fibre bundle that connects the two occipital lobes via the splenium of the corpus callosum. Contributing to the floor of both the occipital horn and the temporal horn is the inferior longitudinal fasciculus at label 6. The inferior longitudinal fasciculus transmits the ventral visual pathway which is responsible for object recognition. Moving dorsally, in the frontal horn at label 7, there is a good example of the superior thalamostriate vein positioned immediately deep to the epithelial lining of the ventricular wall. Label 8 is the anterior perforating substance, an area of mixed grey and white matter that has a high concentration of cholinergic neurons and is perforated by lenticulostriate arteries.	https://sketchfab.com/3d-models/medial-iii-a5dcdd2e37ed44079298baff18685d6d	Fig. 2e
Medial IV, Step 4	The fourth step is to remove the ventricular ependyma from the head and body of the caudate nucleus. Labels 1 and 2 show the head and body of the caudate nucleus respectively. Label 3 marks the point at which the dissector stopped removing the ependymal layer from the caudate nucleus. Note the difference in appearance between the sponge-like body and smooth-like tail. Moving rostro-ventrally, label 4 indicates the olfactory bulb. Caudally, this bulb continues as the olfactory tract. Similarly, from rostral to caudal, labels 6–8 indicate the optic nerve, chiasm, and tract respectively. Due to the level at which the cerebrum was removed from the brainstem, we can appreciate a cross-section through the superior aspect of the midbrain. Label 9 is the red nucleus. This marks the starting point of the rubrospinal tract. Label 10 is the substantia nigra and is a dopaminergic nucleus crucial for motor and movement control. Label 11 indicates the right cerebral peduncle.	https://sketchfab.com/3d-models/medial-iv-47dafa4423204b39b99e50b054785fe7	Fig. 2f
Medial V, Step 5	The last step is to remove the remainder of the ventricular ependyma from the atrium, occipital, and temporal horns of the lateral ventricles. In doing so, the tail of the caudate nucleus is exposed at label (1) Medial to the tail of the caudate nucleus is the stria terminalis at label (2) The stria terminalis carries fibres from the amygdala to the anterior nuclei of the hypothalamus and is thought to play a role in the modulation of autonomic, neuroendocrine, and behavioural responses. The amygdala is labelled number 3 on this scan. Extending dorsally, label 4 indicates the bed nucleus of the stria terminalis. The bed nucleus of the stria terminalis is considered to be an extension of the amygdala due to its role in modulating fear responses. Lastly, label 5 is the stria medullaris, a fibre bundle containing efferent fibres that run from the septal nuclei to the habenula.	https://sketchfab.com/3d-models/medial-v-7bfbc2d8ce434acda58f915f72312095	Fig. 2g

Models are paired with corresponding dissection steps and figures

### Quantitative assessment of mesh complexity and geometric fidelity

Beyond subjective visual inspection, we first compared the resolution of mobile photogrammetric reconstructions with models generated using an open-source high-end photogrammetry pipeline (Vavassori et al. 2025) using mesh density analysis. The number of vertices and faces in each model was automatically quantified using Blender’s statistics panel. Models produced by the high-end pipeline exhibited substantially denser meshes, with an average mesh density three to four times greater than that of the mobile photogrammetry models (Table 2).

Because vertices and face counts are indicators of mesh detail but do not directly measure geometric accuracy, cloud-to-cloud distance analyses using root mean square (RMS) were performed using an open source application software (CloudCompare 2025). RMS quantifies the average deviation of surface points between two 3D models after optical alignment (i.e. iterative closest point registration; ICP). In the case of anatomy, RMS captures both natural biological variability and differences in photogrammetric reconstruction, providing an objective measure of geometric similarity. Lower RMS values indicate closely matching surfaces with minimal variation. Higher values suggest increased deviation or noise.

**Fig. 7:**
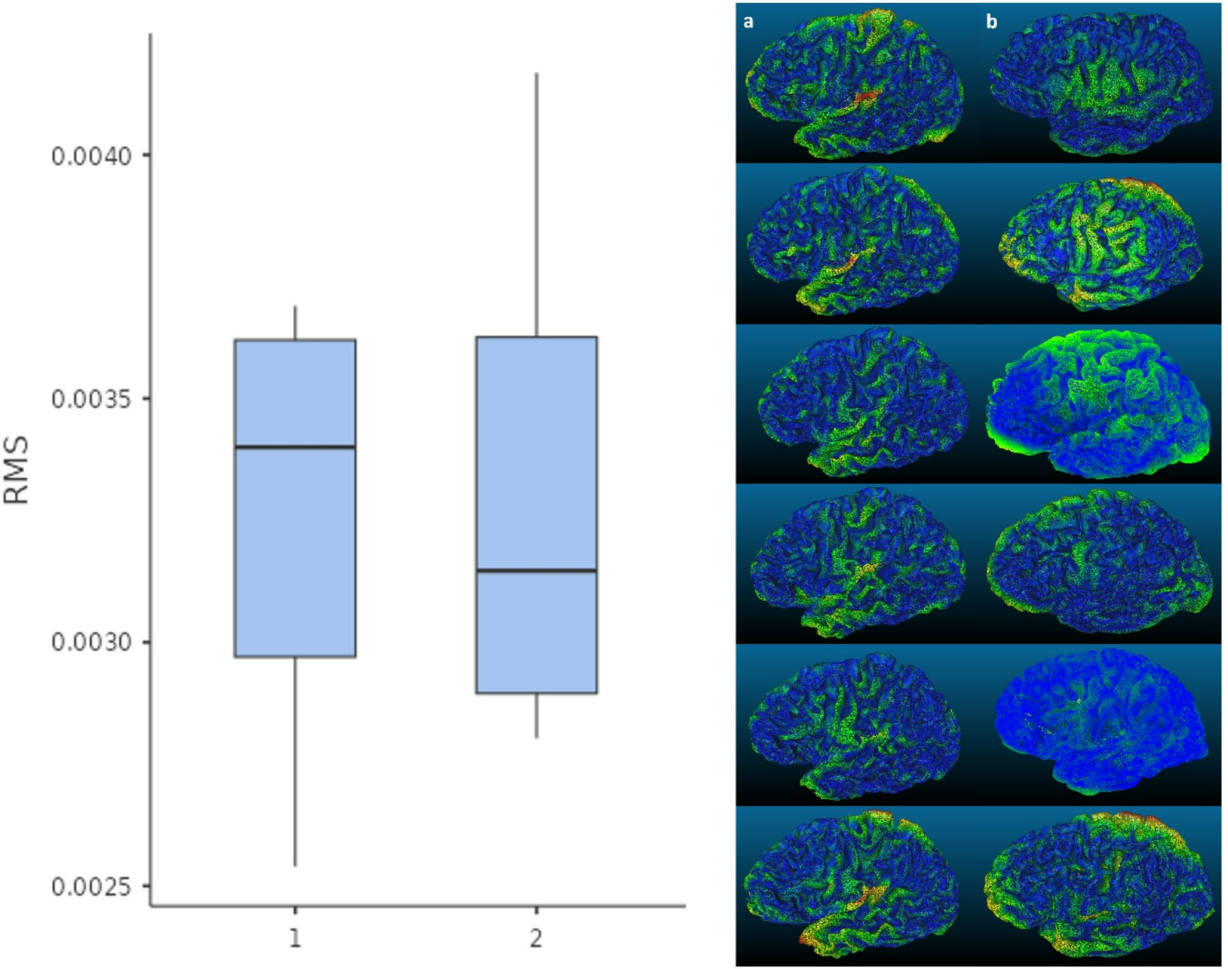
Left, Box and whisker plot for median RMS values for cloud-to-cloud computations where 1 = Mobile vs. High-End group and 2 = High-End S07-E02 vs. High-End group. Right, Colour-coded distance maps of co-registered 3D models. Column a = Mobile vs. High-End heatmaps registered to Lateral I. Images are in numerical order from top to bottom (with Specimen-07 Epoch-02 excluded). Column b = High-End S07-E02 vs. High-End heatmaps using Specimen-07 Epoch-02 as registration model

RMS was computed between the mobile photogrammetry (Lateral I) and six left-lateralised decorticated high-end models with an overall average RMS = 3.26 mm (mean = 2.32 mm, SD = 2.11 mm; Table 3). As a control, RMS was also computed between a randomly selected high-end model (Specimen-17 Epoch-02; S07-E02) and the remaining six high-end models used in the first comparison. Average RMS = 3.31 mm (mean = 2.45 mm, SD = 2.25 mm). An independent samples t-test confirmed no significant difference between the Mobile vs. High-End RMS values and the High-End S07-E02 vs. High-End RMS values (t = − 0.176, df = 10, *p* = 0.568, Cohen’s *d* = − 0.102), demonstrating comparable surface similarity across both comparisons (Fig. 7).

Visual inspection of the cloud-to-cloud distance maps showed that blue regions dominated (Fig. 7). In the Mobile vs. High-End comparisons, yellow to red regions were observed at the insula, dorsal pole, and temporal pole, which may reflect differences in dissection depth rather than reconstruction error. For the High-End S07-E02 vs. High-End comparisons, yellow to red regions appeared at the superior parietal cortex, likely reflecting natural anatomical variation.

**Table 2:** Mesh density metrics for mobile and high-end photogrammetry models with corresponding ratios

Mobile model ID	Density metrics	High-end model ID	Density metrics	Ratio
				Mobile: high-end
Lateral I	V: 163,559	Specimen-02 Epoch-02	V: 468,289	1 : 2.86
	F: 934,539		F: 934,539	1 : 3.47
		Specimen-06 Epoch-02	V: 195,459	1 : 1.19
			F: 387,732	1 : 1.44
		Specimen-07 Epoch-02	V: 698,988	1 : 4.27
			F: 1,390,681	1 : 5.16
		Specimen-10 Epoch-02	V: 447,775	1 : 2.74
			F:888,537	1 : 3.29
		Specimen-15 Epoch-02	V: 347,528	1 : 2.12
			F: 693,205	1 : 2.57
		Specimen-17 Epoch-02	V: 991,154	1 : 6.06
			F: 693,205	1 : 7.34
		Specimen-19 Epoch-02	V: 932,999	1 : 5.70
			F: 1,859,904	1 : 6.91
			Mean V: 583,170	1 : 3.56
			Mean F: 1,161,572	1 : 4.31

Metrics are reported for the mobile ‘Lateral I model’ and descriptively comparative dissections from the high-end pipeline

**Table 3:** Cloud-to-Cloud root mean square computations for mobile and High-End models

Cloud to cloud distance metrics for mobile vs. high-end clouds				Cloud to cloud distance metrics for high-end mobile vs. high end clouds			
Mobile model ID	High-end model ID	Quantitative metrics^†^		High-end model ID	High-end model ID	Quantitative metrics^†^	
Lateral I	Specimen-02 Epoch-02	RMS	0.003304	Specimen-17 Epoch-02	Specimen-02 Epoch-02	RMS	0.003119
		Mean distance	0.02456			Mean distance	0.002195
		SD	0.002087			SD	0.002015
	Specimen-06 Epoch-02	RMS	0.002858		Specimen-06 Epoch-02	RMS	0.002823
		Mean distance	0.001812			Mean distance	0.002045
		SD	0.003664			SD	0.001758
	Specimen-07 Epoch-02	RMS	0.002399		Specimen-07 Epoch-02	RMS	0.004168
		Mean distance	0.002561			Mean distance	0.003015
		SD	0.003498			SD	0.002740
	Specimen-10 Epoch-02	RMS	0.002550		Specimen-10 Epoch-02	RMS	0.003777
		Mean distance	0.002204			Mean distance	0.002610
		SD	0.002204			SD	0.002568
	Specimen-15 Epoch-02	RMS	0.002539		Specimen-15 Epoch-02	RMS	0.003173
		Mean distance	0.001822			Mean distance	0.002221
		SD	0.001545			SD	0.002077
	Specimen-17 Epoch-02*	RMS	0.002894				
		Mean distance	0.002041				
		SD	0.001450				
	Specimen-19 Epoch-02	RMS	0.003694		Specimen-19 Epoch-02	RMS	0.002803
		Mean distance	0.002599			Mean distance	0.002637
		SD	0.002478			SD	0.002317
		Total average RMS	0.003260			Total average RMS	0.003311
		Total average mean	0.002319			Total average mean	0.002454
		Total average SD	0.002115			Total average SD	0.002246

*Not included in total average calculations; used as reference model for high-end S07-E02 vs. high-end computations

^†^Quantitative metrics based on 50000 sample points

## Discussion

This study outlines a cost-effective method for producing models of comparable fidelity to those generated using bespoke equipment. The combined use of the Polycam smartphone application and Blender is one that requires minimal technical expertise and is widely accessible for students, technical staff, and academics in both teaching and research environments. An initial assessment of visual and quantitative reconstruction quality indicates that this workflow is acceptable for educational outputs and can achieve millimetre-level agreement with high-end neuroanatomical photogrammetric models.

Although high-end models were shown to produce much denser meshes, this does not inherently guarantee better educational usability. The models produced in this mobile pipeline, with their lower mesh density but geometric fidelity, might actually be more beneficial in an educational setting for several reasons. For example, lower mesh densities can facilitate smoother real-time manipulation by students who may not have access to top-quality devices or internet connections. Dense meshes can slow rendering and interactivity and introduce unnecessary detail that has no added educational value. For identifying gross anatomical features and understanding structural and spatial orientation, a clean yet moderately detailed mesh may present the ideal and strike a balance between accuracy and usability. Increased mesh density could also be attributable to the variation in dissection technique whereby we removed the cerebral cortex not only from the sulcal concavities but also from the gyral convexities, which should logically decrease the quantity of vertices and faces seen in this study.

These considerations are particularly relevant when viewed against the persistent challenges associated with learning neuroanatomy, a subject frequently identified as a significant contributor of neurophobia (Hazelton 2011; McColgan et al. 2013). Reasons for this point towards topic complexity, inadequate visualisation of prosections, and challenges in appreciating the 3D intricacy of the brain (Javaid et al. 2018). While virtual approaches have become increasingly popular in addressing these challenges (Allen et al. 2016; Aridan et al. 2024; Drapkin et al. 2015; Ekstrand et al. 2018), many have done so at the cost of neuroanatomical realism limiting opportunities to experience anatomical variation; a feature that graphical 3D models and computationally generated fibre representations (e.g. diffusion tensor imaging) cannot fully replicate. In contrast, the use of mobile photogrammetry may be well-placed to curtail the challenges of learning neuroanatomy and strike an appropriate balance between learning the internal topography of the brain in sufficient detail and being able to use resources efficiently in time-limited environments. That said, virtual approaches, including mobile photogrammetry, should be viewed as complementary to rather than replacements for cadaveric dissection wherever possible.

To further contextualise the potential of mobile photogrammetry in education, it is useful to examine the spectrum of photogrammetry workflows and their relative complexities. Photogrammetry is a well-established technical method across diverse scientific fields (Apollonio et al. 2021; Mallison and Wings 2014; Salagean-Mohora et al. 2023; Tsoraeva et al. 2021). In medicine, workflows span elaborate multi-camera systems (De Benedictis et al. 2018; de Oliveira et al. 2023, 2024) and photogrammetric reconstructions of microsurgical photographs (Spiriev et al. 2023) to fully automated smartphone configurations (Morichon et al. 2024; Selvaraj et al. 2022; Trandzhiev et al. 2024), reflecting a wide range of workflow complexities and intended purposes. At the premium end of the scale, de Oliveira et al. (2024) used the MedCreator surface scanner (MedReality, Chicago, IL, US) to generate 3D models of internal brain topography, while Nocerino et al. (2017) employed a dual-camera system with varying image scales, ground sample distances, and focus distances to create dense point clouds for registration with neuroimaging datasets. The diversity in workflow complexity naturally raises questions regarding the consistency of output quality. Thus, an important consideration is whether all readily available photogrammetry smartphone applications produce 3D models of the same standard. While an analysis of how Polycam compares to similar apps is beyond the scope of this study, it is important to acknowledge that the results of smartphone photogrammetry for neuroanatomy may vary depending on the application used, smartphone quality, and LIDAR capability (Habib et al. 2004; Piazza et al. 2024). Moreover, to our knowledge, the use of Polycam in anatomical education and research is yet to be formally documented in the literature, with existing references limited to conference abstracts reporting its application to plastinated specimen of musculoskeletal anatomy (Selvaraj et al. 2022, 2023).

Mobile photogrammetry may also have broader applications in neuroanatomical education and research. Cadaveric brains with both typical anatomy and notable pathology can be preserved and shared with generations of students long after a donor has been interred. The seamless integration of scanning, post-processing, and digital archiving is one that could be adopted by technical officers as part of routine brain extraction and preservation workflows. It is also noteworthy that the FBX files exported from Polycam, along with cropped, edited and converted file types processed in Blender and CloudCompare, are compatible with several platforms including virtual and augmented reality environments. Consequently, with appropriate technical expertise, anatomy departments can develop their own Mixed Reality apps with minimal learning curves.

Lastly, beyond undergraduate teaching and neurosurgical training, greater access to photogrammetric virtual dissections may support researchers engaged in scientific studies of white matter, including those employing tractography and related imaging techniques (Koutsarnakis et al. 2017; Martino et al. 2013; Vavassori et al. 2025). Whether photogrammetric 3D models generated through this current pipeline can be directly integrated with neuroimaging data and registered to standard reference space, as demonstrated in other combined imaging-photogrammetric workflows (De Benedictis et al. 2018; Nocerino et al. 2017), remains an objective for future investigation. Nonetheless, the limited number of heavy outliers or extreme pointwise deviations in this study suggest that such integration with standardised reference spaces is conceptually feasible.

## Conclusion

This study demonstrates the feasibility and benefits of using ubiquitous equipment to generate high-quality 3D models of white matter dissections. Preserving specimen in this manner may enhance neuroanatomy education by offering students an accessible way to engage with and interpret complex spatial relationships within the brain. While the educational outcomes of this approach are not measured here, the projected impacts include improved resource accessibility by providing digital and reusable models of neuroanatomical specimens outside the dissection theatre, enhanced spatial understanding, and practical guidance for educators and developers seeking to create low-cost transferrable teaching resources. The models generated here demonstrate clean topology and congruent surface structure supporting that this workflow can produce geometrically regular 3D surfaces at an educationally useful level of detail without requiring high-end equipment. The study’s strength lies in its stepwise internal repeatability, its affordability, and relative simplicity of its digital workflow, making it an invaluable and accessible approach for enhancing cadaveric neuroanatomy outputs.

### Author contributions

Authors DC, BL, and DB contributed to the study conception and design. Material preparations, data collection and interpretation were performed by DC. The first draft of the manuscript was written by DC and all authors commented on previous versions of the manuscript. All authors read and approved the final manuscript.

### Funding

This work was supported by the Deans Award for Innovation in Teaching 2022-23. Author DB has received research support from Faculty of Health Sciences, Trinity College Dublin.

**Declarations**.

### Conflict of interest

The authors have no relevant financial or non−financial interests to disclose.

### Ethical approval

The human cadaveric brain utilised in this study was sourced from the anatomy donation programme at Trinity College Dublin with consent for research provided at the time of donation. Ethical approval was not required.

## Supplementary Information

Below is the link to the electronic supplementary material.

Supplementary Material 1

## Data Availability

The raw 3D datasets generated during the current study are not publicly available but are available from the corresponding author on reasonable request.
