# Supplementary material for: Immortalising cadaveric brain dissection using application photogrammetry
Source: Brain Struct Funct. 2026 Feb 4;231(2):24. doi: 10.1007/s00429-026-03073-0 (PMC12872721; doi:10.1007/s00429-026-03073-0)
Supplement: Supplementary file 1 — Supplementary Material 1 [file 429_2026_3073_MOESM1_ESM.docx]

# Supplementary File 1

Procedure for quantitatively comparing mobile with high-end photogrammetry models using Blender and Cloud Compare.

Mesh Density Analysis

1. Before exporting the final and edited Polycam models from Blender, the statistics scene statistics panel was consulted. Vertices and faces were recorded. The models were then exported from Blender as .ply files ensuring that vertex normals and triangulated meshes were included.
2. 3D models of a comparative dissection depth (i.e. sulcal decortication) and hemisphere (i.e. left hemisphere) were downloaded from an open-source photogrammetry pipeline (Bradipho) as a high-quality benchmark. These were downloaded as .ply files. These files were first imported to Blender so as to consult vertices and faces.

Cloud-to-cloud Computations (Geometric Fidelity Analysis)

1. Both the Polycam and Bradipho .ply files were imported to Cloud Compare (an open-source 3D viewing and analysis application). Cloud Compare was used to convert the models to point clouds and compute cloud-to-cloud distances analysis.
   1. ***Note:*** *Models from the Bradipho pipeline did not include medial surfaces and were therefore hollow 3D reconstructions. To ensure as fair of a comparison as possible between Polycam and Bradipho reconstructions, the medial surface from the Lateral I model was first removed from the mesh using Blender.*
2. Each mesh was first manually rotated so that meshes roughly corresponded in the same direction and orientation.
3. Each mesh was then individually converted to a point cloud (Select model -> Edit -> Mesh -> Sample points). Points number was set to 1,000,000.
4. IPC alignment was then performed (Tools -> Registration -> Fine Registration [ICP]). We aligned our Polycam Lateral I Point Cloud to each of the Bradipho Point Clouds as reference.
5. Cloud-to-cloud computations were then performed (Tools -> Distances -> Cloud/Cloud Dist). The role assigned to the Polycam cloud was ‘Compared’. The role assigned to the Bradipho clouds was ‘Reference’. Distance computation settings were set to default. The console panel gave the mean distance and standard deviation point distances.
6. After computing the cloud-to-cloud distances, an overlaid reference image was produced.
